# Expression profiles and prognostic values of BolA family members in ovarian cancer

**DOI:** 10.1186/s13048-021-00821-0

**Published:** 2021-06-02

**Authors:** Mingyang Zhu, Shiqi Xiao

**Affiliations:** Department of Nursing, Shengjing Hospital of China Medical University, No.36 Sanhao Street, Shenyang, Liaoning 110004 People’s Republic of China

**Keywords:** BolA family members, Ovarian cancer, Prognosis, Database, Bioinformatics analysis

## Abstract

**Background:**

The BOLA gene family, comprising three members, is mainly involved in regulating intracellular iron homeostasis. Emerging evidence suggests that BolA family member 2 plays a vital role in tumorigenesis and hepatic cellular carcinoma progression. However, there was less known about its role in ovarian cancer.

**Methods:**

In the present study, we investigated the expression profiles, prognostic roles, and genetic alterations of three BolA family members in patients with ovarian cancer through several public databases, containing Oncomine and Gene Expression Profiling Interactive Analysis, Human Protein Atlas, Kaplan–Meier plotter and cBioPortal. Then, we constructed the protein-protein interaction networks of BOLA proteins and their interactors by using the String database and Cytoscape software. In addition, we performed the Gene Ontology (GO) and Kyoto Encyclopedia of Genes and Genomes (KEGG) pathway enrichment by the Annotation, Visualization, and Integrated Discovery database. Finally, we explored the mechanisms underlying BolA family members’ involvement in OC by using gene set enrichment analysis.

**Results:**

The mRNA and protein expression levels of BOLA2 and BOLA3 were heavily higher in ovarian cancer tissues than in normal ovarian tissues. Dysregulated mRNA expressions of three BolA family members were significantly associated with prognosis in overall or subgroup analysis. Moreover, genetic alterations also occurred in three BolA family members in ovarian cancer. GO analysis indicated that BolA family members might regulate the function of metal ion binding and protein disulfide oxidoreductase activity. Gene set enrichment analysis indicated that BolA family members were mainly associated with oxidative phosphorylation, proteasome, protein export, and glutathione metabolism in ovarian cancer.

**Conclusion:**

In brief, our finding may contribute to increasing currently limited prognostic biomarkers and treatment options for ovarian cancer.

## Introduction

Ovarian cancer (OC) is a gynecological malignant tumor ranking fifth leading cause of cancer-related death in women [27]. Approximately 295,414 cases newly diagnosed, and 184,799 cases died in 2018 [3]. Most patients are diagnosed at an advanced stage due to OC’s lack of apparent symptoms in early-stage and inadequate predictive biomarkers, leading to a shallow 5-year survival rate [6, 20]. Despite diagnosis and treatment in developed countries recently, OC’s survival rates improved little for decades. Therefore, exploring gene signatures related to OC progression and prognosis and identifying new biomarkers for predicting prognosis and directing OC’s treatment strategies is a crucial clinical challenge of critical significance.

The BolA gene family contains BolA family member 1 (BOLA1), BOLA2, and BOLA3 and widely conserved crosswise Gram-negative bacteria and eukaryotes [13]. Previous studies showed BOLA proteins were mainly linked to stress response, iron homeostasis, and iron-sulfur cluster assembly and trafficking [17]. Recent studies showed BOLA2 played critical roles in the biology and prognosis of hepatic cellular carcinoma (HCC) [12, 19]. However, little knowledge about the function of the BolA gene family in other cancers.

In the present study, we analyzed the expression and prognostic values of BolA family members in OC by using online databases to provide potential prognostic biomarkers and new individualized targets for OC.

## Materials and methods

Oncomine database was used for the analysis of the expression patterns of BOLA members in OC. The differential expression of BOLA members between normal controls and OC samples was performed by Oncomine database (www.oncomine.org), an online cancer microarray database and web-based data-mining platform [25]. The search contents and thresholds were set as follows: keywords, BOLA1, BOLA2 and BOLA3, primary filter, cancer vs. normal; cancer type, OC, sample type, clinical specimen; data type, mRNA; the absolute value of fold change > 2, *P* < 0.05; and gene rank, 10%. The student’s t-test was used to calculate the *P*-value.

### GEPIA and HPA database analysis for the validation

The Gene Expression Profiling Interactive Analysis (GEPIA) database (http://gepia.cancer-pku.cn/), a new web-based tool, supplies an online analysis of data from The Cancer Genome Atlas (TCGA) [32]. In our study, we used it to perform the differential mRNA expression analysis of BolA family genes between OC samples and normal controls (|Log2FC| cutoff = 1; *p*-value cutoff = 0.05), differential mRNA expression analysis of BolA family genes in different pathological stages, and correlation analysis between the expression BolA family genes. The Human Protein Atlas (https://www.proteinatlas.org/) offers tremendous amounts of transcriptomics, proteomics data, and IHC-based protein expression patterns in specific normal human tissues compared with tumor tissues [18]. In the present study, we systematically screen the available immunohistochemistry images of BolA family proteins presented in the database. Then typical images were selected to show the different expressions in normal ovarian tissues and OC tissues.

### The Kaplan–Meier plotter analysis of the prognostic value of BOLAs in OC

The Kaplan–Meier plotter (http://kmplot.com/analysis) is an online database that can be used to evaluate the values of 54,675 genes in survival rates of ovarian, breast, lung, and gastric cancer patients [7–9, 30]. In the present study, we analyze the prognostic significance of BolA family genes in OC patients. The selected OC samples were split into two groups based on the auto-selected best cutoff. Three BolA family members (BOLA1, BOLA2, and BOLA3) were put into the database to acquire Kaplan-Meier survival plots, and all the datasets were used, with 1656 patients for OS and 1435 patients for PFS. The subgroup survival analysis according to histology, grade, stage, TP53 mutation status, applied chemotherapy was performed. The hazard ratio (HR) with 95% confidence intervals (CIs) and log-rank *P* values were calculated and listed in survival plots. *P* < 0.05 was considered statistically significant.

### cBioPortal database analysis of genomic alteration of BOLAs in OC

The cBioPortal for Cancer Genomics (http://cbioportal.org), an open-access web resource, offers multidimensional cancer genomic data from TCGA [5]. In the present study, one TCGA dataset of OC named TCGA Provisional (606 cases) was selected to be further analyzed for BOLAs gene mutations or copy number alterations (CNA) in OC. The InfoPrint, co-expression, mutations, and survival tabs were applied according to the online instructions of the cBioPortal.

### GO and PPI analysis for function and interaction of BOLAs in OC

The gene-gene interaction of the BolA family gene and their interactors network was built via the Gene Multiple Association Network Integration Algorithm (GeneMANIA; https://www.genemania.org). The Search Tool for the Retrieval of Interacting Genes Database (STRING v.10.0; https://string-db.org/)was used to set up a protein-protein interaction (PPI) network [31, 34]. The Cytoscape software was utilized to conceive network graphs for PPI analysis [28]. Enrichment analysis of Gene Ontology (GO) and Kyoto Encyclopedia of Genes and Genomes (KEGG) pathway of BolA family genes and their interactors were explored via the Database for Annotation, Visualization, and Integrated Discovery (DAVID; v.6.8; https://david.ncifcrf.gov/home.jsp) [10].

### Gene set enrichment analysis (GSEA)

To explore the possible mechanism associated with BOLA members’ involvement in OC’s carcinogenesis, we performed GSEA by GSEA program from sangerbox software (http://sangerbox.com/) to find out the pathways connected to the diverse BOLA expression in the TCGA OC tissues [29]. The annotated gene set file c2.cp.kegg.v6.0.symbols. GMT (from the MSig database) was used for reference. Normalized enrichment score (NES) > +/− 2, nominal *P* value (NOM *P*-Val) < 0.05 and false discovery rate (FDR) < 0.05 were evaluated for statistical significance.

## Results

### Expression profiles of BolA family members in OC

**Fig. 1:**
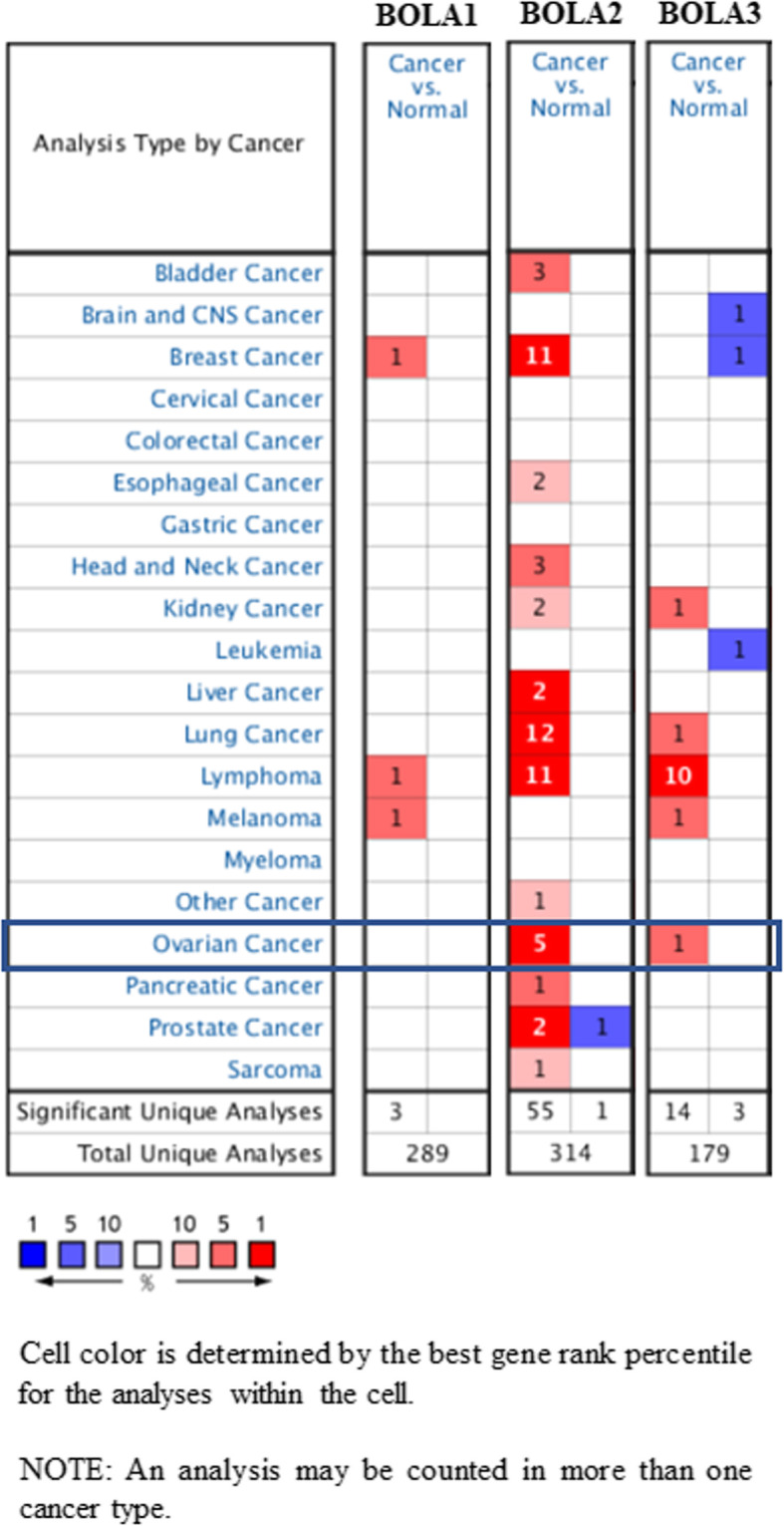
Expression levels of BOLA family members in different types of cancers (Oncomine). Each cell’s number represents the number of analyses conformed to the following threshold: *P* < 0.05, the absolute value of fold change > 2, and gene rank, 10%. The numbers in colored cells show the quantities of datasets with statistically significant mRNA higher expression (red) or lower expression (blue) of BOLA family members

We firstly analyzed dysregulated transcriptional levels of BolA family members in 20 various types of human cancers in the Oncomine database. As listed in Fig. 1, BolA family members might act as either tumor promoters or suppressors in diverse kinds of tumors. In overall OC patients, the mRNA expression levels of BOLA1 and BOLA2 were significantly upregulated than those in normal ovarian tissues in Bonome’s dataset with a fold change of 1.373 and 3.545, respectively. However, no data on the differential mRNA expression of BOLA3 in overall OC tissues than normal ovarian tissues, as shown in Table 1. We also summarized the mRNA levels of BolA family members in different OC types obtained from Oncomine datasets in Table 1. For BOLA1, the mRNA expression level was significantly higher in various kinds of ovarian cancer tissues than normal ovarian tissues in Lu′s dataset. Concerning BOLA2, the mRNA expressions were higher in some kinds of ovarian cancer tissues in Lu′s dataset. However, they were higher in various kinds of ovarian cancer tissues than normal ovarian tissues in Hendrix’s dataset. As to BOLA3, the mRNA expression level was higher in some kinds of ovarian cancer tissues than in normal ovarian tissues.

**Table 1:** The significant changes of BOLA family members’ expression between different types of OC and normal tissues (Oncomine)

BOLA	Types of OC vs. Normal															Ref/Source
	Ovarian Carcinoma			Endometrioid			Clear cell			Serous			Mucinous			
	FC	***P***	N	FC	***P***	N	FC	***P***	N	FC	***P***	N	FC	***P***	N	
**BOLA1**	1.373	**< 0.001**	195	–	–	–	–	–	–	–	–	–	–	–	–	Bonomeovarian
	–	–	–	1.268	**< 0.001**	9	1.191	**0.004**	7	1.180	**0.001**	20	1.096	**0.027**	9	Lu ovarian
	–	–	–	1.032	0.709	37	1.093	0.912	8	1.005	0.468	41	1.072	0.867	13	Hendrix ovarian
**BOLA2**	3.545	**< 0.001**	195	–	–	–	–	–	–	–	–	–	–	–	–	Bonome ovarian
	–	–	–	2.098	**< 0.001**	9	2.083	**< 0.001**	7	2.166	**< 0.001**	20	–	–	–	Lu ovarian
	–	–	–	1.196	**0.003**	37	1.228	**0.002**	8	1.191	**0.003**	41	1.191	**0.005**	13	Hendrix ovarian
**BOLA3**	–	–	–	2.003	**0.001**	9	1.455	0.065	7	1.537	**< 0.001**	20	1.490	**0.024**	9	Lu ovarian

The bold font indicates the difference between OC and normal tissues conformedtothe selected thresholds

*OC* ovarian cancer, *FC* fold change, “–” not available, *N* number of patients

**Fig. 2:**
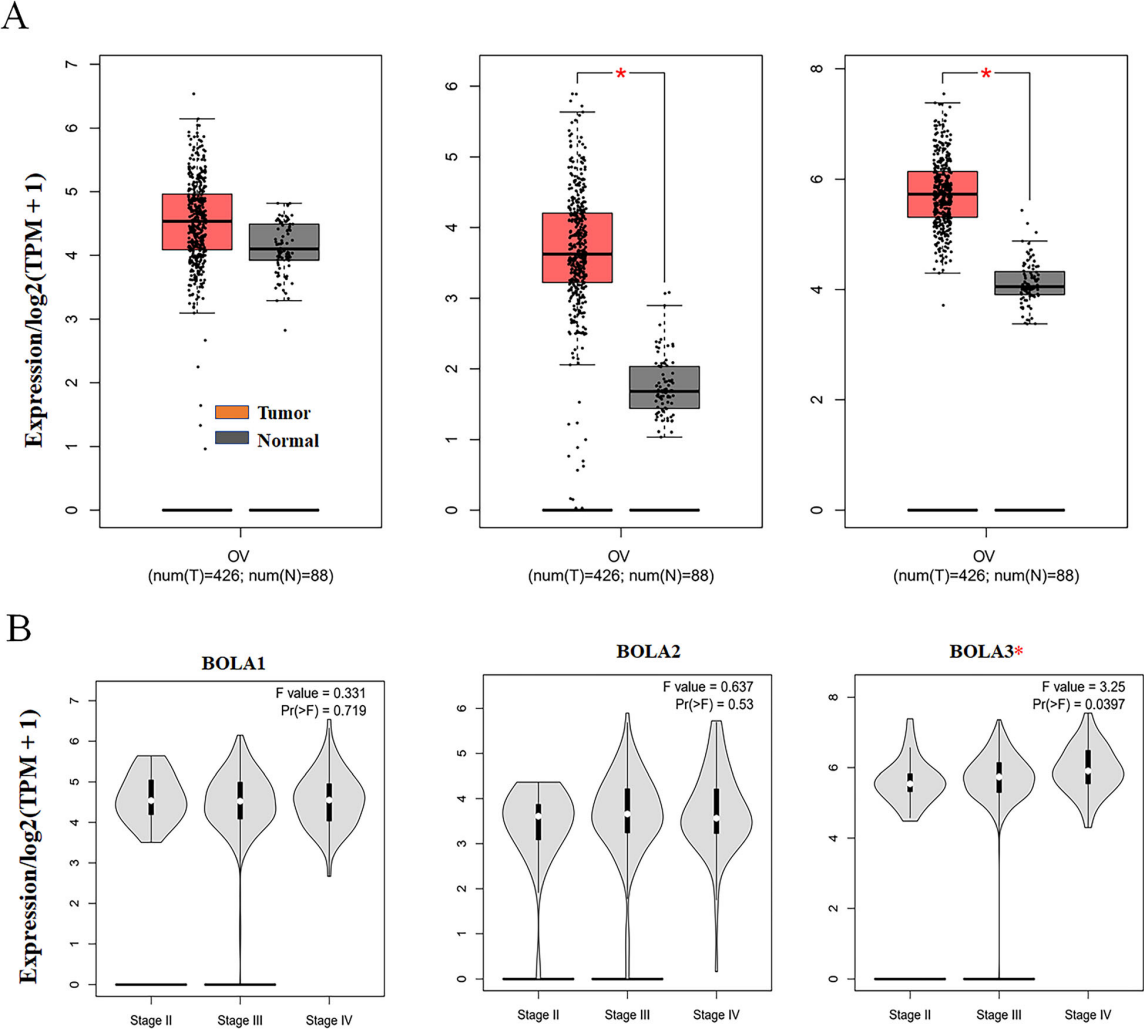
Dysregulated mRNA expression levels of BOLA family and the association with tumor stage in OC. **a** Box plots of dysregulated mRNA expression BOLA family members in OC based on GEPIA database. **b** The relationship between mRNA expression of BOLA family members and tumor stages in OC patients

Additionally, the GEPIA database was utilized to contrast the differential mRNA expression of BolA family members between OC and normal ovarian tissues. As shown in Fig. 2a, the expression levels of BOLA2 and BOLA3 were remarkably higher, and the mRNA level of BOLA1 was slightly upregulated (*P* > 0.05) in OC tissues than normal ovarian tissues, which corresponded with results of the Oncomine database except for that of BOLA1. Moreover, the correlation between mRNA expression levels of BolA family members and different OC stages was also analyzed, and only BOLA3 was significantly upregulated in the higher stage (Fig. 2b).

**Fig. 3:**
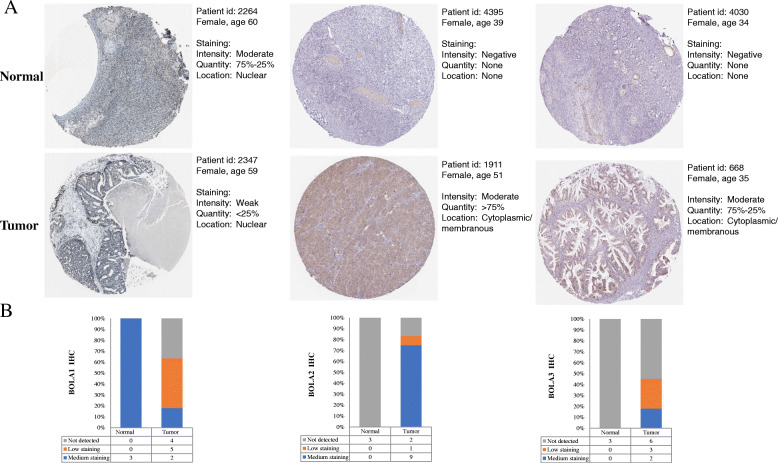
The protein expression profile of BOLA family members in regular ovarian compared with cancer tissues. Representative immunohistochemistry images (**a**) and expression status (**b**) of BOLA family members in normal ovarian tissues and ovarian cancer tissues based on HPA database (http://www.proteinatlas.org/)

Moreover, we analyzed BolA family members’ protein expression in normal ovarian tissues and OC tissues using the HPA database. As shown in Fig. 3, we found that ovarian stroma cells had medium BOLA1 staining in 3 cases of normal ovarian tissues. Relatively, among 11 cases of OC tissues examined, 2 cases had medium BOLA2 staining, 5 cases had low BOLA2 staining, and 4 cases had no BOLA1 staining. For BOLA2, it was undetected in normal ovarian tissues, however, among the examined 12 OC tissues, 9 cases had medium BOLA2 staining, 1 case had low BOLA2 staining, and 2 cases had no BOLA2 staining. For BOLA3, the data showed no BOLA3 staining in normal ovarian tissues. In comparison, among 11 cases of OC tissues examined, there were 2 cases of mediumBOLA3 staining, 3 cases of low BOLA3 staining, and 6 cases of no BOLA3 staining.

So, these results indicated BolA family members might function as oncogenes in OC and maybe a possible therapeutic target of precision therapy for patients with OC.

### Prognostic value of BolA family members in patients with OC

**Fig. 4:**
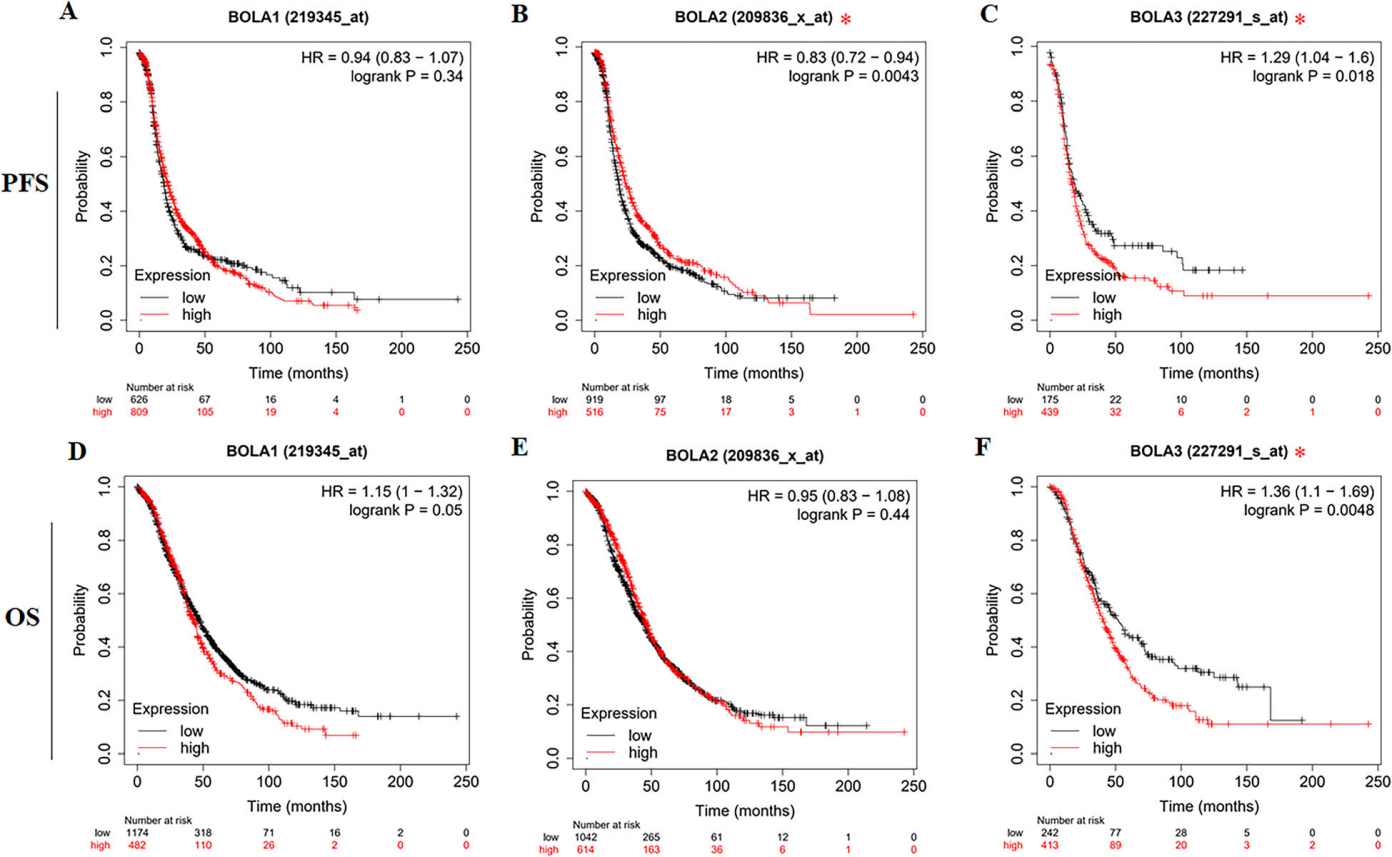
Prognostic values of mRNA level of BOLA family members in OC patients. Kaplan-Meier curves show the correlation between mRNA expression of BOLA family members and progression-free survival (**a–c**) and overall survival (**d–f**) of OC patients. * *P* < 0.05

We firstly appraised the relationship between the mRNA expression of BolA family members and the survival in all OC patients via Kaplan-Meier plotter analysis. All the datasets were used, with 1656 patients for OS and 1435 patients for PFS. The data demonstrated that the increased BOLA3 mRNA level was associated with shorter progression-free survival (PFS) for overall survival (OS) of OC patients, while decreased BOLA2 mRNA level was associated with shorter PFS of OC patients (Fig. 4). However, there was no relation between BOLA3 mRNA level and the prognosis of OC patients.

Then, we also assessed the prognostic values of BolA family members in different subtypes of OC patients defined according to different histology, clinical stages, pathological grades, and TP53 status by Kaplan–Meier plotter analysis. As shown in Table 2, increased mRNA expression of BOLA3 was significantly related to shorter OS in serous OC patients for different histology. For clinical stages, low mRNA expression of BOLA2 predicted poor OS in stage 2, low mRNA expression of BOLA3 was related to shorter OS in stage 4, while high mRNA expression of BOLA3 predicted the poor OS in stage 3. In terms of pathological grades, high BOLA1 mRNA expression was linked to the poor OS in grades 1–2, while high mRNA expression of BOLA2 and BOLA3 predicted favorable OS in grade 3. Interestingly, increased expression of BOLA2 predicted favorable OS in mutated TP53 type, while increased expression of BOLA1was associated with longer OS in wild-type TP53.

**Table 2:** The relationship between BOLA family members and OS in other different subtypes of OC (Kaplan-Meier plotter)

Subtypes		BOLA1			BOLA2			BOLA3		
		Cases	HR (95% CI)	***P value***	Cases	HR (95% CI)	***P value***	Cases	HR (95% CI)	***Pvalue***
Histology	Serous	1207	0.93(0.8–1.08)	0.34	1207	0.86(0.73–1.01)	0.061	523	1.36(1.04–1.77)	**0.022**
	Endometrioid	37	2.13(0.24–19.05)	0.49	37	4.42(0.74–26.51)	0.075	30	3.77(0.39–36.42)	0.22
Stage	1	74	2.29 (0.73–7.25)	0.15	74	0.43 (0.14–1.35)	0.14	51	2.52 (0.6–10.55)	0.19
	2	61	0.52 (0.18–1.5)	0.22	61	0.28 (0.08–1.01)	**0.038**	32	0.16 (0.02–1.36)	0.057
	3	1044	1.15 (0.97–1.36)	0.097	1044	1.15 (0.95–1.38)	0.15	426	1.38 (1.05–1.81)	**0.019**
	4	176	1.38 (0.95–2.01)	0.087	176	0.69 (0.47–1.02)	0.059	61	0.49 (0.26–0.9)	**0.019**
Grade	1 + 2	380	1.54 (1.16–2.06)	**0.003**	380	0.79 (0.6–1.06)	0.11	203	1.54 (0.97–2.46)	0.064
	3	1015	1.11 (0.92–1.34)	0.28	1015	0.82 (0.7–0.98)	**0.025**	392	0.73 (0.56–0.96)	**0.022**
TP53	Mutated	506	0.84 (0.65–1.08)	0.17	506	0.71 (0.56–0.89)	**0.0025**	124	0.79 (0.54–1.16)	0.23
	WT	94	0.5 (0.29–0.89)	**0.015**	94	0.54 (0.25–1.14)	0.1	19	1.38 (0.49–3.92)	0.54
CR	Platin	1409	1.14 (0.99–1.32)	0.07	1409	0.94 (0.81–1.09)	0.41	478	1.30 (1–1.69)	**0.047**
	Taxol	793	1.16 (0.95–1.4)	0.14	793	1.17 (0.97–1.41)	0.11	357	1.39 (1.01–1.93)	**0.043**
	Taxol + platin	776	1.17 (0.97–1.43)	0.10	776	1.19 (0.98–1.44)	0.075	356	1.40(1.01–1.93)	**0.041**

The bold font indicates the difference was significant statistically

*OC* ovarian cancer, *OS* overall survival, *WT* wild type, *CR* Chemotherapy Regimen

As referred to PFS (Table 3), increased mRNA expression of BOLA3 was significantly related to shorter PFS in serous OC patients, and decreased levels of BOLA2 predicted inferior PFS in serous OC patients. For clinical stages, high mRNA expression of BOLA2 was connected to longer PFS in stage 1, stage 2, stage 3, and stage 4, high mRNA expression of BOLA1 predicted longer PFS in stage 3, whereas low levels of BOLA1 predicted longer PFS in stage 4. For pathological grades, increased levels of BOLA2 predicted better PFS in all grades, high mRNA expression of BOLA1 and BOLA2 were remarkably related to shorter PFS in grades 1–2. In comparison, low expression of BOLA3 predicted shorter PFS. Moreover, increased expression of BOLA2 was correlated with inferior PFS in OC patients either with mutated TP53 or wild type.

**Table 3:** The relationship between BOLA family members and PFS in other different subtypes of OC (Kaplan-Meier plotter)

Subtypes		BOLA1			BOLA2			BOLA3		
		Cases	HR (95% CI)	***P value***	Cases	HR (95% CI)	***P value***	Cases	HR (95% CI)	***Pvalue***
Histology	Serous	483	0.93(0.81–1.08)	0.33	483	0.8(0.69–0.93)	**0.0048**	483	1.36(1.08–1.72)	**0.0091**
	Endometrioid	44	2.19(0.84–5.7)	0.10	44	2.05(0.81–5.21)	0.12	44	1.95(0.44–8.72)	0.37
Stage	1	96	1.72 (0.59–5.01)	0.32	96	0.27 (0.1–0.79)	**0.01**	74	0.62 (0.17–2.2)	0.46
	2	67	1.6 (0.69–3.73)	0.27	67	0.29 (0.12–0.72)	**0.0047**	41	1.94 (0.56–6.73)	0.29
	3	919	0.82 (0.71–0.96)	**0.013**	919	0.81(0.7–0.95)	**0.0083**	424	1.21 (0.95–1.53)	0.13
	4	162	1.52 (1.04–2.24)	**0.031**	162	0.59 (0.38–0.92)	**0.018**	70	0.60 (0.34–1.07)	0.082
Grade	1 + 2	293	1.48 (1.04–2.1)	**0.028**	293	0.69 (0.52–0.92)	**0.0098**	189	1.66 (1.13–2.44)	**0.0085**
	3	837	1.11 (0.92–1.34)	0.3	837	0.85 (0.72–1)	**0.049**	315	0.74 (0.55–0.99)	**0.04**
TP53	Mutated	483	1.24 (0.99–1.56)	0.065	483	0.7 (0.56–0.88)	**0.0018**	124	1.30 (0.87–1.94)	0.19
	WT	84	0.62 (0.36–1.06)	0.077	84	0.54 (0.31–0.95)	**0.031**	19	0.60(0.21–1.73)	0.34
CR	Platin	1259	0.85 (0.75–0.97)	**0.018**	1259	0.79 (0.69–0.91)	**< 0.001**	502	0.81 (0.65–1)	0.054
	Taxol	715	0.87 (0.74–1.04)	0.12	715	0.8 (0.67–0.95)	**0.011**	381	1.30(1–1.69)	**0.046**
	Taxol + platin	698	0.89 (0.75–1.06)	0.2	698	0.81 (0.68–0.97)	**0.021**	380	1.301–1.69)	**0.049**

The bold font indicates the difference was significant statistically

*OC* ovarian cancer, *PFS* progression-free survival, *WT* wild type, *CR* Chemotherapy Regimen

In general, these results suggested that the mRNA expression levels of BolA family members could be considered optional biomarkers for predicting OC patients’ survival.

### Genomic alteration of BolA family genes in OC

**Fig. 5:**
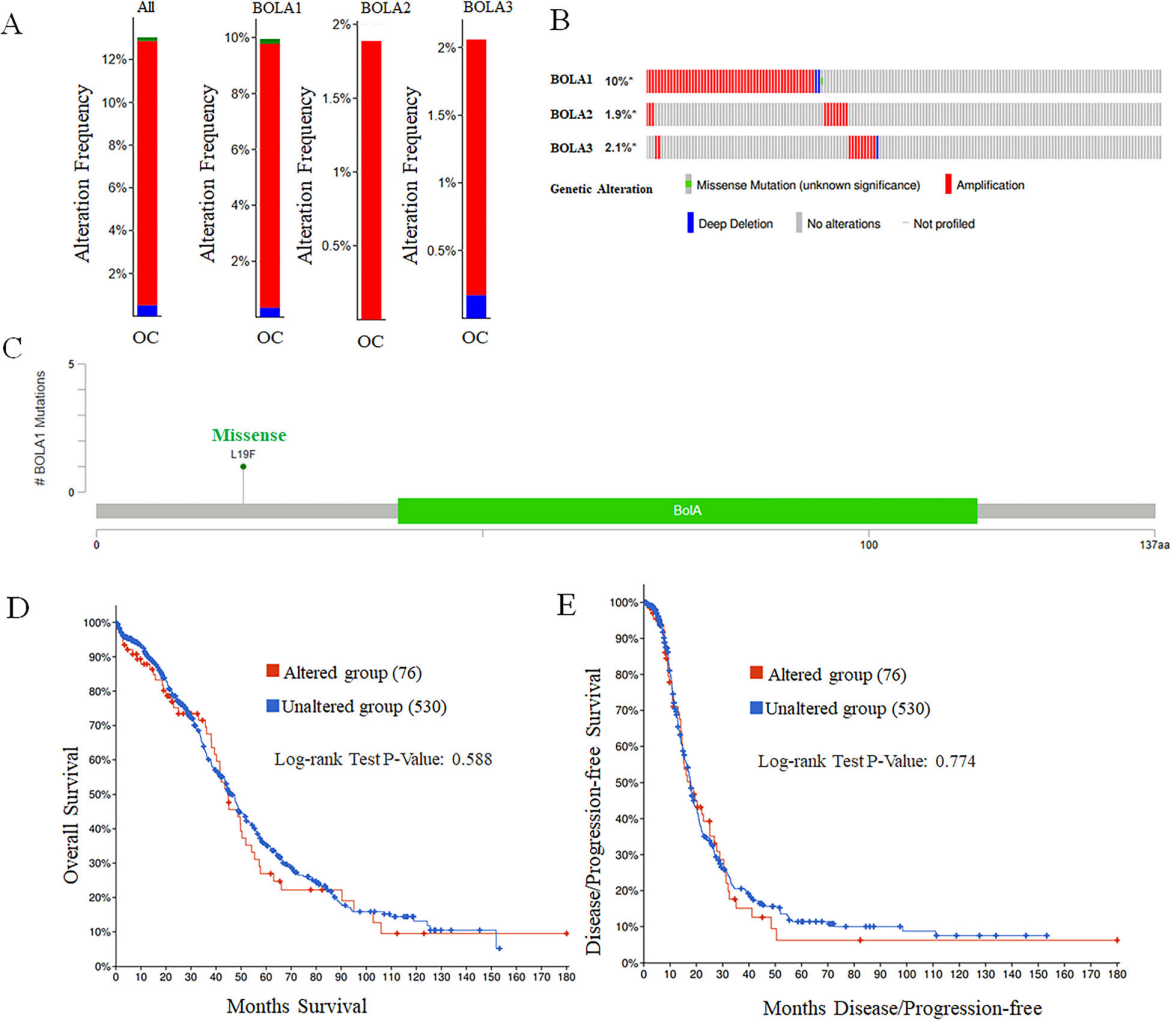
Genomic analysis of BOLA family members in OC based on cBioPortal databases. **a** Summary of genomic alteration of BOLA family members in OC. **b** Oncoprint visual summary of alteration on a query of BOLA family members in OC. **c** The mutations of BOLA1 were plotted. Kaplan–Meier plots comparing **d** overall survival (OS) and **e** progression-free survival (PFS) in cases with/without BOLA family members’ gene alterations

We then explored the possible mechanisms involved in the dysregulation of BolA family members’ expression in OC. We analyzed the genetic alteration frequency of BolA family members using the cBioPortal online tool for OC (The Cancer Genome Atlas, Provisional). Six hundred six patients were analyzed totally. The monoprints included missense mutation, deletion, and amplification, with the ratios of genetic alterations of BolA family members in OC varied from 1.89 to 9.95% (Fig. 5a, b). The ratios of genetic mutation in BOLA1, BOLA2, BOLA3 were 9.95% (0.17% mutation, 9.43% amplification, 0.34 deep deletion), 1.89% (amplification), 2.06% (1.89%amplification, 0.17% mutation) respectively. Besides, missense mutation was identified in BOLA1(Fig. 5c). Moreover, the survival curves indicated that cases with or without alterations in one of the BOLAs were not related to OS and PFS (Fig. 5c, d) using the Kaplan–Meier plot analysis and log-rank test.

### Function and interaction of BolA family members

**Fig. 6:**
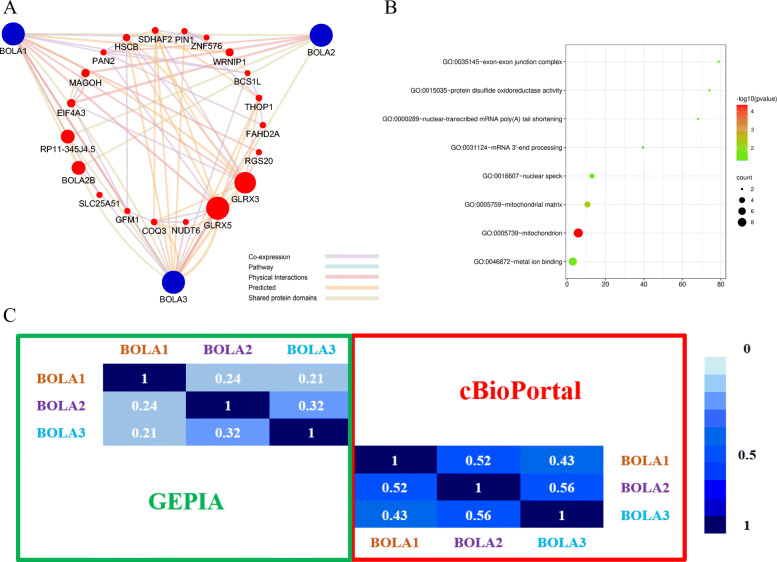
Protein-protein interaction (PPI) and function enrichment of BOLA family members. **a** The network of 3 BOLA family members and 20 proteins significantly interacted with BOLA family members (String). **b** Gene Ontology (GO) enrichment analysis of BOLA family members and their interactors (DAVID). **c** The Pearson correlation coefficients between BOLA family members based on GEPIA and cBioPortal databases

A network of three BolA family members and 20 kinds of proteins associated with BolA family members was set up using the String database and Cytoscape software. The network showed that BolA family members were associated with several metal-ion binding-related genes such as glutaredoxin 5 (GLRX5), glutaredoxin 3 (GLRX3), Werner helicase interacting protein 1 (WRNIP1), Etc. (Fig. 6a). Next, GO enrichment and KEGG pathway analysis of BolA family members and their interactors were conducted using DAVID. We found that the BolA family members were mainly related to mitochondrion and mitochondrion matrix location. It might exert its functions by targeting metal ion binding and protein disulfide oxidoreductase activity (Fig. 6b). Finally, the Pearson correlation coefficients were calculated between BolA family members using correlation analysis in GEPIA and cBioPortal databases, ranging from 0.21 to 0.56 (Fig. 6c).

### GSEA identifies BOLAs-regulated pathways in OC

**Fig. 7:**
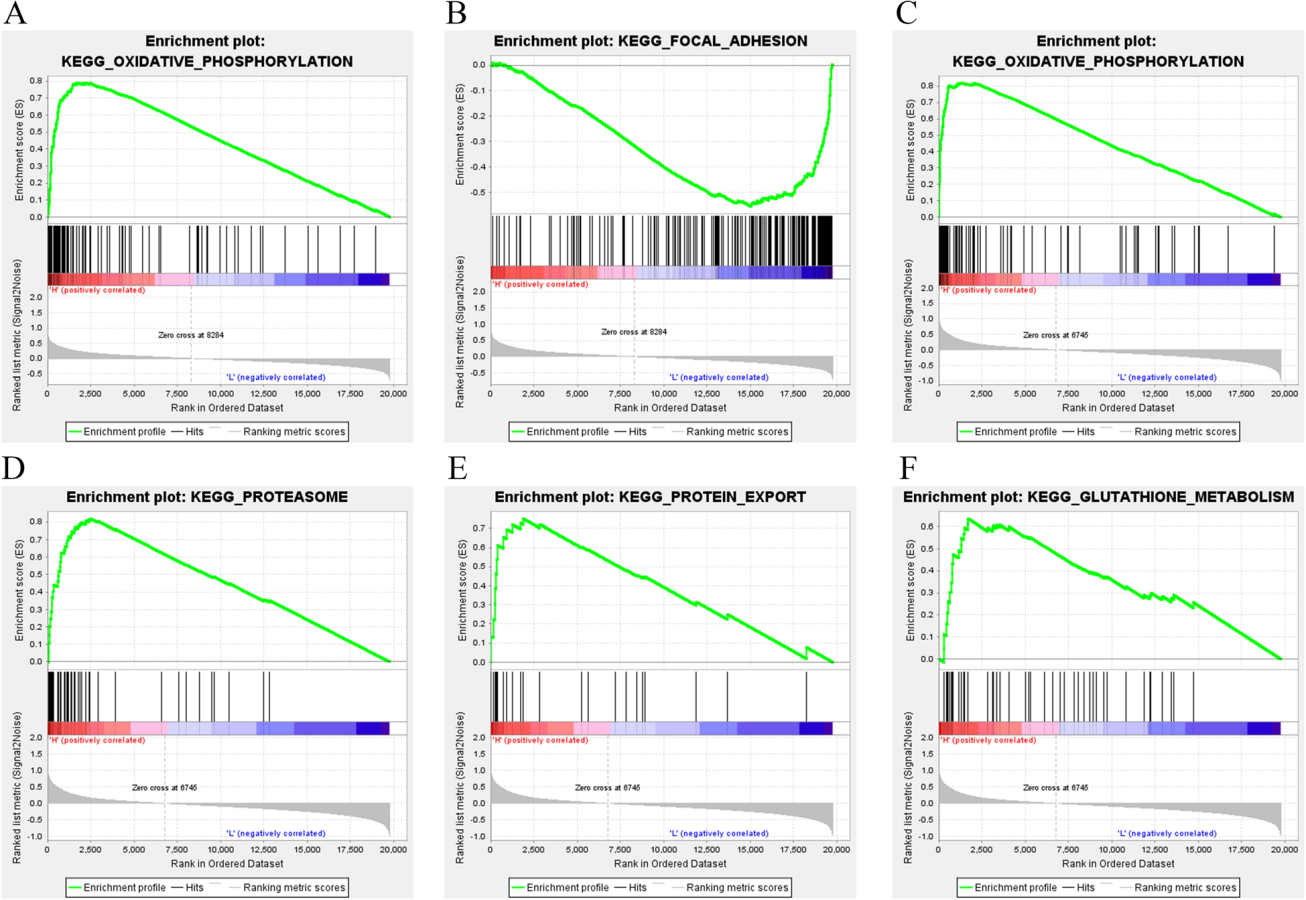
Gene set enrichment analysis (GSEA) analysis of BOLA family members. **a** GSEA showed that BOLA1 expression was positively correlated with oxidative phosphorylation. **b** GSEA showed that BOLA1 expression was negatively correlated with the focal adhesion. GSEA showed that BOLA3 expression was positively correlated with oxidative phosphorylation (**c**), proteasome (**d**), proteins export (**e**), and glutathione metabolism (**f**) in OC

To investigate the alteration of BOLA-related pathways in OC, GSEA analysis in OC with high or low expression levels of each BOLA gene was performed, gene sets with a Normalized Enrichment Score (21) > +/− 2, FDR < 0.05, and *p* < 0.05 were identified as the hallmark gene sets, as shown in Fig. 7. In the pathway enrichment analysis, a high expression of BOLA1 was positively correlated with several oxidative phosphorylations (Fig. 7a) while negatively correlated with the focal adhesion in OC (Fig. 7b). High expression of BOLA3 expression was positively correlated with oxidative phosphorylation, proteasome, protein export, and glutathione metabolism in OC (Fig. 7c, d, e, f). However, there was no hallmark gene sets enrichment for high or low BOLA2 expression in OC.

## Discussion

Currently, the prognostic of patients with OC remains poor and could be attributed to the lack of valuable biomarkers for early diagnosis, prognosis evaluation, and precision therapy. Hence, it is vital to learn about the gene signatures associated with OC’s genesis and development to pick out new molecular markers for early diagnosis, target therapy, and evaluating prognosis. Former studies have reported that BolA family members functions as vital regulatory factors for intracellular iron homeostasis in the micro-environment [4, 17]. More and more evidence indicated that iron homeostasis dysregulation was connected to oncogenesis and development [2]. In recent years, accumulated pieces of evidence have suggested BOLA2 was involved in HCC occurrence and development. In contrast, BOLA gene family members’ expression and clinical relevance in OC patients were still unclear.

We explored the expression feature of the BolA family members in OC using a series of bioinformatics methods to identify the potential targets for accurate therapy. Our data demonstrated that the expression of BOLA1, BOLA2, and BOLA3 in human OC tissues were all notably higher than normal ovarian tissues in the Oncomine database. In contrast, BOLA2 and BOLA3 in human OC tissues were much higher than normal ovarian tissue in the GEPIA database. Next, we found the protein expression of BOLA2 and BOLA3 were in human OC tissues was significantly higher than that in normal ovarian tissues in the HPA database, which was almost consistent with mRNA expression data of the Oncomine database and GEPIA database. Our data indicated that BOLA2 and BOLA3 might be the potential targets for accurate therapy for OC patients.

We next explored the prognostic values of the BolA family members in OC by using Kaplan-Meier online plotter database. Our data showed the increased BOLA3 mRNA level was correlated with shorter PFS and OS, while decreased BOLA2 mRNA level was associated with shorter PFS. In subgroup analysis, we further found that abnormal BOLA1 expression was closely related to OC patients’ prognosis with histological grade G1–2, TP53 wild-type, or stage 3–4, respectively. For BOLA2, subgroup analysis showed abnormal mRNA expression was closely related to the OC patients’ prognosis with serous pathological subtype, histological grade G1–3, TP53 wild-type, TP53 mutation, or stage 1–4, respectively. For BOLA3, abnormal mRNA expression was closely associated with OC patients’ prognosis with serous pathological subtype, histological grade G1–3, or stage 3–4, respectively. These results indicated that BOLA1, BOLA2, and BOLA3 might be new prognostic biomarkers for OC patients.

Next, to predict the potential mechanisms of BOLAs in the progression and prognosis of OC, we constructed the PPI network by using three BOLA members and 20 of their interactors and found BOLAs were associated with several metal-ion binding -related genes, including GLRX5, GLRX3, WRNIP1, ZNF576, THOP1, FAHD2A, and HSCB. Previous studies showed GLRX3, GLRX5, WRNIP1, THOP1, and HSCB were all regulators of various cancer occurrence and progression [14–16, 24, 33]. Our data and present studies showed that BolA family members might mediate OC’s progression and prognosis by interacting with these metal-ion binding-related genes. We further investigated the alteration of BOLAs-related pathways in OC and found BOLA1 was mainly related to the focal adhesion and oxidative phosphorylation in OC. BOLA3 was mainly related to oxidative phosphorylation, proteasome, protein export, and glutathione metabolism in OC. Prevenient studies have reported that focal adhesions’ coordinated and dynamic regulation is needed for cell migration, essential in cancer metastasis [11, 22]. Other studies have indicated oxidative phosphorylation, proteasome, protein export, and glutathione metabolism are all involved in cancer progression [1, 21, 23, 26]. Our data and previous studies showed that BolA family members might promote OC’s progression and prognosis by affecting these pathways, and the precise mechanism needs to clarify.

Finally, there are still some limitations in our study. For one thing, the clinical data available in these databases are finite, and data of several essential factors, including chemotherapy resistance, CA125 level, lymph node metastasis, and tumor size that may affect OC’s prognosis, were missing. The correlation between protein expression of the BolA family and OC prognosis is not clear. Thirdly, the precise mechanism of the BolA family members’ impact on OC patient prognosis has not been addressed. Lastly, systematic tests on the expressions, roles, and prognostic value of the BolA family have not been investigated.

## Conclusions

The present comprehensive bioinformatic analysis clarified that BOLA1, BOLA2, and BOLA3 might be optional prognostic biomarkers, and BOLA2 and BOLA3 may be a possible therapeutic targets for precision therapy for patients with OC. Nevertheless, further experimental studies are urgently needed. Our finding may contribute to increasing limited prognostic biomarkers and treatment options for ovarian cancer.

## Data Availability

Several publicly available datasets were used in this study, and these data can be found on the following websites: www.oncomine.org, http://gepia.cancer-pku.cn/, http://kmplot.com/analysis/, https://www.cbioportal.org/, https://string-db.org/, https://david.ncifcrf.gov/.

## Abbreviations

OC: Ovarian cancer
BolA: Bovine major histocompatibility complex
BOLA: BolA family member
HCC: Hepatic cellular carcinoma
TCGA: The Cancer Genome Atlas
GEPIA: Gene Expression Profiling Interactive Analysis
HR: Hazard ratio
CIs: Confidence intervals
CNA: Copy number alterations
GeneMANIA: Gene Multiple Association Network Integration Algorithm
PPI: protein-protein interaction
GO: Gene Ontology
KEGG: Kyoto Encyclopedia of Genes and Genomes
DAVID: Database for Annotation, Visualization, and Integrated Discovery
GSEA: Gene set enrichment analysis
NES: Normalized enrichment score
FDR: False discovery rate
PFS: Progression-free survival
OS: For overall survival
